# Substance use among Ghanaian adolescents in secondary education: the determinants and medico-social implications

**DOI:** 10.1186/s12889-025-22647-x

**Published:** 2025-05-02

**Authors:** Walter Appati, Easmon Otupiri, Adelaide Appati, Eliezer Bernard Owusu Ntim

**Affiliations:** 1 Department of Internal Medicine, Komfo Anokye Teaching Hospital, Kumasi, Ghana; 2 Department of Public Health, Kwame Nkrumah University of Science and Technology, Kumasi, Ghana; 3 Department of Obstetrics and Gynaecology, Komfo Anokye Teaching Hospital, Kumasi, Ghana; 4 Department of Psychiatry, Komfo Anokye Teaching Hospital, Kumasi, Ghana

**Keywords:** Substance use, Determinants, Medico-social implications, Adolescents, Secondary education

## Abstract

**Background:**

A significant canker that has been with us for some years now and continues to be on the ascendency, especially among the youth in Ghana, is the use of substances. Adolescence is a period where adolescents tend to indulge in various behaviours, including substance use, as a result of vulnerability to societal influences.

**Methods:**

A cross-sectional descriptive study was carried out in the New Juaben Municipality, Ghana, to ascertain the determinants of substance use and its medico-social implications on selected second-cycle students. Two hundred and forty (240) students between 12 and 19 were sampled to participate in the study and interviewed using interviewer-guided pretested questionnaires.

**Results:**

The mean age for substance use was found to be 17.80 years. The majority began substance use as early as between 16–18 years (79.5%). The males (67.5%) indulged in substance use more than the females (32.5%). Most of the students have used alcohol before (53.2%), followed by marijuana (wee) (19.2%) and tobacco (cigarette) (10.9%). Curiosity (53.9%) and peer influence (33.3%) are the main factors driving substance use initiation among students. Majority (39.7%) of students experienced an inability to study for tests, while 29.5% got into fights under the influence of substances. However, the majority (78.2%) of students affirmed their willingness to receive help if addicted to any substance.

**Conclusion:**

Establishing peer groups and counselling units in secondary institutions can provide crucial support for students, particularly those struggling with addiction, mitigating potential medical and social consequences.

**Supplementary Information:**

The online version contains supplementary material available at 10.1186/s12889-025-22647-x.

## Background

In 2021, it was globally estimated that the use of substances increased from 240 million in 2011 to 296 million (5.8% of the global population). This population was people aged between 15 and 64 [1].

Meanwhile, a study conducted in Ghana revealed that, out of the overall number of 50,000 substance users in Ghana, 35,000 were aged between 12 and 35, while the remaining 15,000 were adults, made up of 9,000 males and 6,000 females. Research from fifteen (15) psychiatric hospitals in Ghana revealed that about 70% of inmates in those hospitals were youth from various educational institutions [2].

Substance use, including alcohol, marijuana (wee) and tobacco (cigarette) (10.9%), is a universal problem for youngsters, affecting health, social, economic, cognitive, and humanity [3]. The majority of the research conducted suggested that early to late adolescence is a high-risk period for substance initiation, and it may be pivotal among young people [1, 4].

Sometimes, women reported to have substance use disorders do have a previous history of post-traumatic stress disorder, with attendant challenges during childhood, such as repetitive abuse or neglect [4]. Studies have revealed that males who have encountered childhood distress use drugs as a means of social defiance. At the same time, females who have experienced adversity are more likely to internalise it as anxiety, depression and social withdrawal [7].

The traditional gender gap of substance use being higher among males than females seems to still predominantly exist, with a lifetime prevalence rate significantly greater for boys compared to girls [4, 8].

While the harmful use of alcohol resulting in chronic health consequences may not result in disability until later in life, acute health consequences have grave effects on the youth [9]. The probable risk factors of psychoactive substance use included school levels, gender, age groups, and friends and family members smoking. Adolescence, therefore, is generally a vulnerable period where most adolescents initiate various behaviours, including the use of substances [10].

The overall risk or protective factors in a child's life are an interplay of the interactions between the individual's intrinsic characteristics and their various life experiences. A poor child-parent bond in infancy may lead to early behavioural problems, which can retard performance at school and relationships with peers. There is a call for a well-coordinated, comprehensive, and rights-based approach to strengthen children and young people's ability to harness protective factors while addressing risk factors [11].

Analysing some research findings revealed that substance use is predominant among adolescents from broken homes in Ghana [12]. Adolescents may already have acquired drinking and smoking habits, and when family problems arise, they may drink and/or smoke more and more [10]. However, another study revealed that there is no absolute relationship between family and substance use, emphasising that not all adolescents growing up in families with these risk factors end up becoming illicit drug users [13].

It is widely known that substances such as tobacco, alcohol, cannabis, and various allopathic drugs are used by many young people, especially those in schools around the world [5]. During the developmental periods of adolescence, the brain is intrinsically more susceptible than a mature brain to the adverse long-term effects of environmental insults, such as exposure to tetrahydrocannabinol [6].

Researchers found that drunkenness, tobacco, and other drug use increase the likelihood of engaging in sex as well as having multiple sexual partners. The engagement in such behaviour increases the susceptibility to sexually transmitted infections and heightens the risk of teenage pregnancy and teenage parenthood [3].

Substance use among adolescents is an area of unremitting concern because of the amplified potential consequences on adolescent neurological and social development [14]. As evidenced by available data, substance use among children and young people globally has been associated with numerous negative consequences for education, including school dropout, poor academic performance, and incompletion of education [15, 16].

Understanding the psychosocial and behavioural components of substance users will help to reduce the risk caused as a result of substance use and the potential loss of their lives and careers, as well as to their families and society as a whole.

The study objectives were to ascertain the knowledge on substances most commonly used by Senior High School (SHS) students, determine the risk and protective factors associated with substance use in these populations, evaluate implications for prevention, and assess the perceived effects of substance use on the individual and society in general.

## Methods

### Study design and population

A cross-sectional descriptive design, which involved quantitative approaches in the form of questionnaires, was employed to explore substance use among students in selected second-cycle institutions. Second-cycle institutions, which provide education between basic elementary and tertiary education, are called Senior High Schools (SHS) in Ghana. We considered a probability (random) sampling of students of both genders from Senior High School grade 1 (SHS 1) to Senior High School grade 3 (SHS 3) pursuing various courses at two (2) second-cycle institutions in the New Juaben Municipality, Ghana.

Both institutions sampled comprise males and females with access to day and boarding facilities. Programmes offered include General Science, General Arts, Business, Home Economics, Technical, and Visual Arts.

### Sampling method

These institutions were mainly chosen because of headline-making stories concerning the suspension of nineteen (19) students who indulged in substance use on campus [17]. Another reason for their consideration was based on reports that students sneak out of the school without permission and escape punishment from school authorities [18]. This act exposes students to all forms of immorality, including substance use. The two institutions were selected based on a non-probability (purposive) sampling, while the students were randomly sampled from the institutions.

### Inclusion and exclusion criteria

Participants currently enrolled in a Senior High School (SHS) and aged between 12 and 19 were allowed to participate. Only students who voluntarily agreed to take part in the study were included. Students below or above the target age, i.e., those younger than 12 or older than 19, were excluded. Students with known cognitive impairments or severe psychological disorders that could affect their ability to provide reliable responses were excluded.

### Sampling size

This study employed a sample size of 240, drawn from students between 12 and 19 at both second-cycle institutions.

Based on a random sampling, the sample size for the population was determined using Cochrane’s formula given as: ${n}_{0}=\frac{{Z}^{2}p (1-p)}{{e}^{2}}$, where (${n}_{0}$) is the desired sample size, e is the desired level of precision (i.e., margin of error) at 5% = 0.05, z is the expected standard deviation at 95% CI = 1.96, p is the (estimated) proportion of the population which has the attribute in question = 0.19, q = 1-p as the proportion who do not use substances (1–0.19 = 0.81).

Therefore, ${n}_{0}=\frac{{1.96}^{2}(0.19) (0.81)}{{0.05}^{2}}$. The result was 236.5, with an allowance for errors and non-responses, so the final sample size was 240 respondents.

### Sampling techniques

The students' daily attendance register was used in the sampling process until the sample size was reached. The students in each course class were divided by 6 to obtain the sampling interval. With this, the first subject was randomly chosen, while the succeeding subjects were also selected using the predetermined sampling intervals. Hence, a minimum of 8 students in 29 course classes, equal to 232 respondents, was used, and the questionnaires were successfully distributed to 240 participants.

### Data collection techniques and tools

Structured questionnaires on the research topic were available to the second-cycle institutions' sampled respondents. We used closed-ended questions to elicit specific responses. The questionnaires were designed based on the variables of interest (Appendix). The questionnaires had four sections comprising demographic background, knowledge of substances commonly used, risk and protective factors for substance use, and implications of substance use.

### Data management and analysis

The principal statistical methodology utilised in the research was frequency distribution per the objectives. The questionnaires were cross-checked thoroughly and entered into IBM SPSS Statistics 27 and Microsoft Excel 2019. We analysed the gathered data, generated responses, and used cross-tabulations, charts, logistic regression and Pearson's Chi-Square test to determine associations between studied variables and substance usage. Odds ratios (OR) with 95% confidence interval (CI) were also used to measure associations. All *p*-values less than 0.05 at a significant level of 5% were considered significant. The dependent variable was substance use.

### Ethical considerations

Ethical clearance for the study was obtained from the institution’s Internal Review Board (SMS/CoH/DRP/Vol 1), and another clearance was given by the New Juaben Municipal Health Directorate (MHD/NJ/0621) asking permission from authorities of the selected schools. The Parent Teacher Association (PTA), who act as proxy guardians in the institutions, gave informed consent, while students assented to participate in the study. We ensured that the students had anonymity to prevent linking the data to any particular student due to our high confidentiality and privacy approach to issues regarding substance use.

## Results

### Introduction

Two hundred forty students from both second-cycle institutions, comprising 162 males (67.5%) and 78 females (32.5%), responded to the questionnaire.

### Demographic background and substance use

The mean age of the 240 participants was 17.31 years. Students who have used substances before had a mean age of 17.80 years, whereas students who have not used substances before had a mean age of 17.17 years. 156 (65.0%) students have used substances before, while 84 (35.0%) students have never used substances. From the respondents, a total of 124 (79.5%) students began substance use as early as 16–18 years, with a total of 26 (16.7%) students starting substance use at 13–15 years. 6 (3.8%) students used substances for the first time between 10–12 years. There was a significant difference (*p* = 0.002) in the age of substance use initiation between those who had used substances before and those who denied substance usage.

Most respondents were males (162, 67.5%), with 78 (32.5%) females. Of the population that uses substances, 114 (73.1%) male students have used substances before, and 42 (26.9%) female students attested to using substances before. The test result showed no significant difference (*p* = 0.175) in substance use between males and females.

Regarding the level of academic status, 47 (19.6%) students were in SHS 1, 49 (20.4%) students were in SHS 2, and 144 (60.0%) students were in SHS 3. Of the population that uses substances, 21 (13.5%) SHS 1 students agreed to have used substances before. 38 (24.3%) SHS 2 students also subscribed to the fact that they have used substances before. SHS 3 students recorded a total of 97 (62.2%) students who have used substances. The outcome of this test concerning the use of substances among the level of academic status was significant (*p* = 0.031).

Regarding residential status, 205 (85.4%) students were in boarding, with 35 (14.6%) students being day students. Among the students in boarding, 132 (84.6%) students agreed that they had used substances before, while 24 (15.4%) day students also attested to substance usage. The test result revealed no significant difference (*p* = 0.822) between boarding and day students in the use of substances (Table 1).

**Table 1:** Demographic background and substance use

Variable	Total population (*N* = 240)	Substance use (*N* = 156)	Χ^2^ (chi-square)	*P*-value
**Age**				
Mean age (years)	17.31	17.80	—	—
**Age of initiation**				
10–12 years	31 (12.9%)	6 (3.8%)	**12.45**	**0.002**
13–15 years	57 (23.8%)	26 (16.7%)		
16–18 years	152 (63.3%)	124 (79.5%)		
**Sex**				
Male	162 (67.5%)	114 (73.1%)	**1.84**	**0.175**
Female	78 (32.5%)	42 (26.9%)		
**Academic status**				
SHS 1	47 (19.6%)	21 (13.5%)	**6.92**	**0.031**
SHS 2	49 (20.4%)	38 (24.3%)		
SHS 3	144 (60.0%)	97 (62.2%)		
**Residential status**				
Boarding	205(85.4%)	132 (84.6%)	**0.05**	**0.822**
Day	35 (14.6%)	24 (15.4%)		

However, regression analysis can deduce that age is a significant predictor (*p* = 0.021), with an odds ratio (OR = 1.24), meaning that with each additional year, the odds of substance use increase by 24%. Also, compared to individuals who started substance use at 10–12 years, those who initiated at 13–15 years were 2.84 times more likely to use substances (*p* = 0.009), and those who started at 16–18 years were 10.15 times more likely (*p* < 0.001). Although males (OR = 1.44) had a higher likelihood of substance use than females, this association was not statistically significant (*p* = 0.198).

SHS 2 students were 2.19 times more likely than SHS 1 students to use substances (*p* = 0.024), while SHS 3 students were 2.83 times more likely than SHS 1 students (*p* = 0.012). This suggests that students in higher grades have increased odds of engaging in substance use. Boarding students had slightly lower odds of substance use (OR = 0.94) compared to day students, but the difference was not statistically significant (*p* = 0.820) (Table 2).

**Table 2:** Logistic regression results for substance use

Variable	Β (Coefficient)	Odds ratio (OR)	95% CI	*P*-value
**Intercept**	-3.812	—	—	0.002
**Age**	0.214	1.24	1.03–1.49	0.021
**Age of initiation (Ref: 10–12 years)**				
13–15 years	1.045	2.84	1.30–6.23	0.009
16–18 years	2.317	10.15	3.46–29.74	< 0.001
**Sex (Male = 1)**	0.365	1.44	0.83–2.50	0.198
**Academic status (Ref: SHS 1)**				
SHS 2	0.782	2.19	1.11–4.30	0.024
SHS 3	1.041	2.83	1.26–6.32	0.012
**Residential status (Boarding = 1)**	-0.058	0.94	0.45–1.98	0.820

### Knowledge of substances commonly used

**Fig. 1:**
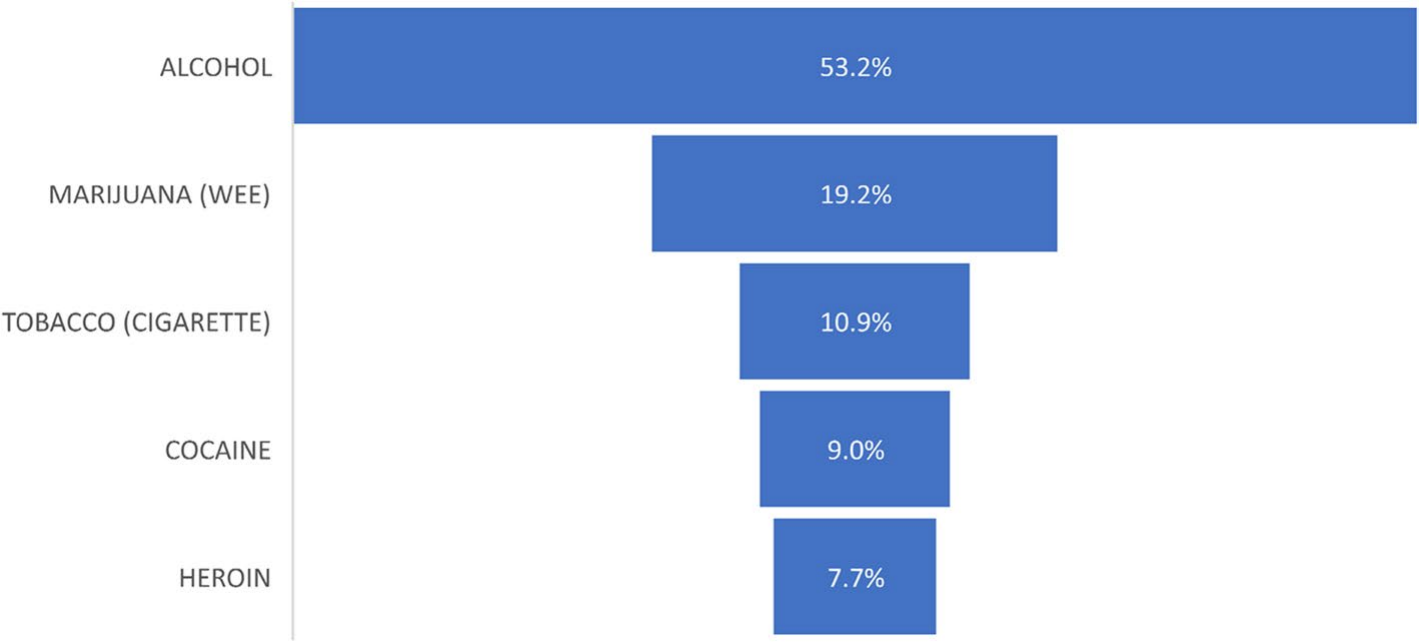
Substances commonly used

Analysing the substances which the students have used before, the majority of the students (83, 53.2%) had used alcohol before, while 12 (7.7%) students had used heroin before. 17 (10.9%) students have a history of tobacco (cigarette) use, with 30 (19.2%) students also having used marijuana (wee) before. Students who indicated they had used cocaine before were 14 (9.0%) (Fig. 1).

Regarding those who introduced the individual to the substances, most (117, 75.0%) of the population ascribed it to friends, while 8 (5.1%) students pointed it out to their family members. However, 14 (9.0%) students indicated they were introduced to substances by drug pushers, with 17 (10.9%) students introduced to substances by social media.

### Risk and protective factors for substance use

**Fig. 2:**
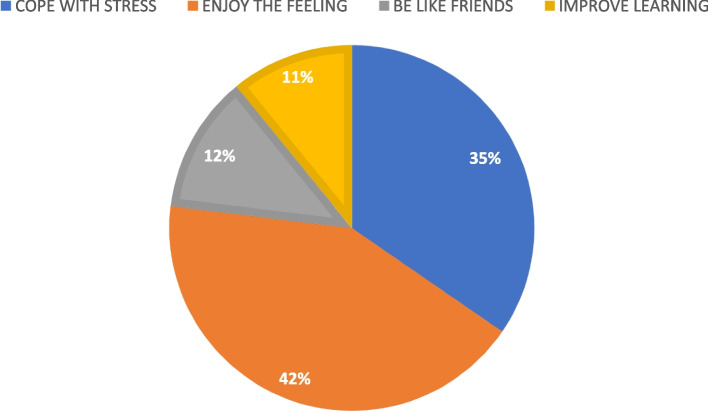
Reasons for substance use

Regarding the main reasons for substance use, the majority (66, 42.3%) of the students said they enjoyed the feeling. 54 (34.6%) students stated it was a coping mechanism for stress, while 19 (12.2%) students used substances to be like their friends. 17 (10.9%) students also used substances to improve learning (Fig. 2).

The majority (84, 53.9%) of students attributed their first substance use to curiosity, while 12 (7.7%) students used their first substance due to parents' or relatives' offers. Those who first used substances because friends encouraged them were 52 (33.3%), with 8 (5.1%) students using substances for the first time to get away from their problems.

### Implications of substance use

With the issue of being unable to do homework or study for a test as a result of substance use, 62 (39.7%) students indicated they had experienced it, with 46 (29.5%) students getting into a fight under the influence of substances. 11 (7.1%) students indicated they caused shame and embarrassment to someone under the influence of substance. Also, 12 (7.7%) students stated neglect of duties and responsibilities due to substance use.

**Fig. 3:**
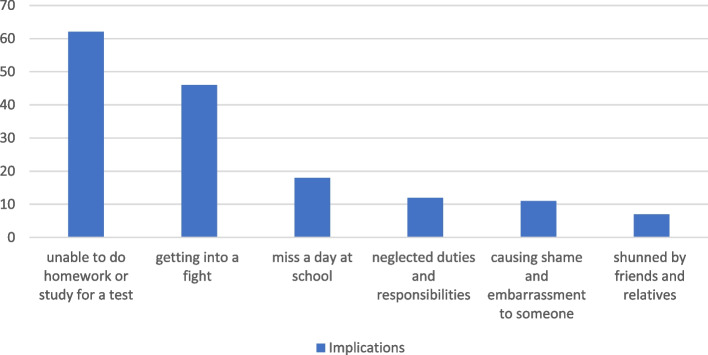
Implications of substance use

7 (4.5%) students indicated friends and relatives shunned them due to substance use, while 18 (11.5%) students indicated missing a day at school under the influence of substances. The outcome of this test showed a significant difference (*p* = 0.0015) between those who had used substances before and those who denied so concerning the implications encountered (Fig. 3).

The Chi-Square Statistic (Χ2) is 97.77, with a *p*-value of < 0.001. Since the *p*-value is extremely small (< 0.05), this suggests that there is a significant association between substance use and the reported negative implications. These findings reinforce that substance use among students is strongly linked to adverse academic, social, and emotional consequences. Intervention programs should focus on raising awareness of these adverse outcomes to discourage substance use.

From this study, 122 (78.2%) students were willing to seek help if addicted to any substance, while 34 (21.8%) students expressed their unwillingness to seek help if addicted to any substance.

Assessing the knowledge of the students on the medical consequences of substance use, the majority (58, 37.2%) of the students were of the view that it can cause mental illness. 19 (12.2%) students also asserted it can lead to stroke, with 28 (17.9%) students thinking substance use can cause heart disease. 51 (32.7%) students also were of the view that substance use can culminate in lung diseases.

## Discussion

This study provides valuable insights into the prevalence and knowledge of commonly used substances among Senior High School (SHS) students, determine the risk and protective factors associated with substance use in these populations, and assess the perceived effects of substance use.

### Prevalence of substance use

The findings indicate a mean participant age of 17.31 years, with those reporting substance use averaging slightly older at 17.80 years. The results further confirm that the majority (79.5%) of the individuals initiated substance use between 16–18 years. Older students and those who initiated substance use at later ages had significantly higher odds of being substance users. These trends are consistent with existing research, which highlights adolescence as a critical period for substance use initiation, primarily due to developmental and psychosocial factors that increase susceptibility to experimentation and risk-taking behaviours [4].

Although sex and residential status were not significantly associated with substance use, males and day students had slightly higher odds of engaging in substance use. More males (73.1%) involved in substance use than females (26.9%), reflecting widely documented gender disparities that suggest males are more likely to engage in risk-taking behaviours, including substance use, compared to females [16]. The slightly higher prevalence among boarding students (84.6%) compared to day students (15.4%) suggests that reduced parental supervision and peer influences in boarding schools may contribute to increased substance use. This finding contrasts with some studies that report higher substance use among day students, possibly due to greater environmental exposure [19]. This highlights the importance of strengthening school-based monitoring and intervention strategies within boarding schools.

Additionally, substance use was observed to increase as students progressed through school, with SHS 3 students recording the highest prevalence (62.2%). This trend underscores the potential impact of academic pressures, social stressors, and greater autonomy in final-year students, reinforcing the need for targeted interventions addressing stress management and healthy coping strategies [20]. The positive association between academic level and substance use underscores the need for targeted intervention programs that address stress management and mental health support among high school students.

### Knowledge of commonly used substances

Alcohol was identified as the most commonly used substance (53.2%), followed by marijuana (19.2%) and tobacco (10.9%). This pattern aligns with prior findings that alcohol is the most widely consumed substance among adolescents, often serving as a gateway to the use of other substances [21]. The relatively high prevalence of alcohol use raises concerns about its accessibility, normalisation in social settings, and underestimation of associated risks. Given its well-documented impact on impulse control, academic performance, and risk-taking behaviours, alcohol consumption among adolescents warrants urgent public health interventions.

The substantial proportion of students who reported marijuana and tobacco use highlights an emerging challenge, particularly considering the growing perception of marijuana as a low-risk substance. This trend emphasises the need for comprehensive drug education programs that address misconceptions and provide evidence-based awareness of the long-term health effects of substance use.

### Risk and protective factors associated with substance use

The findings of this study highlight curiosity (53.9%) and peer influence (33.3%) as the two main factors driving substance use initiation among students. Adolescents are particularly vulnerable to social and environmental pressures, and curiosity often serves as a primary motivator for experimentation [22]. The desire for social belonging during adolescence also plays a crucial role, with peer influence serving as a strong determinant of risk behaviours, including substance use. These findings are consistent with the Social Learning Theory, which suggests that adolescents are more likely to adopt behaviours they observe being reinforced within their social circles [8].

While risk factors were prevalent, protective factors such as awareness of health risks emerged as potential deterrents. A significant proportion of students demonstrated knowledge of the medical consequences of substance use, with 37.2% associating it with mental illness. In contrast, others recognised risks including lung disease, heart disease, and stroke [23, 24]. This level of awareness suggests that educational initiatives on substance-related health risks are somewhat effective. Still, there remains a need for enhanced, evidence-based interventions that focus on both the short-term and long-term health risks associated with substance use.

### Perceived effects of substance use

The study further examined the perceived effects of substance use on academic performance and social behaviour. A substantial proportion (39.7%) of students reported that substance use negatively impacted their ability to complete homework or study for tests, while 29.5% admitted getting into fights under the influence. These findings support existing literature indicating that substance use is strongly associated with cognitive impairments, decreased academic performance, and increased aggression [7, 25]. The academic consequences of substance use suggest that schools should integrate substance abuse prevention into mental health and educational support services.

The study also revealed that most students were aware of the severe health risks associated with substance use, including its potential to cause mental illness, lung diseases, heart disease, and stroke [26]. This level of awareness is encouraging but also highlights a critical gap between knowledge and behaviour, as many students who recognised the dangers of substance use continued to engage in it. This underscores the need for more interactive and behaviourally focused educational programs that go beyond knowledge dissemination to address the psychological and social drivers of substance use.

Encouragingly, 78.2% of students expressed willingness to seek help if addicted to substances. However, 21.8% indicated reluctance to seek assistance, likely due to stigma, fear of punishment, or lack of access to support services. Research has consistently shown that stigma surrounding substance use disorders impedes individuals from seeking help, thereby increasing the risk of long-term dependency and adverse health outcomes [27]. This underscores the need for destigmatisation campaigns and confidential counselling services within schools to facilitate early intervention and support for students struggling with substance use.

## Limitation

The method of data collection involved self-disclosure of substance use. Since issues concerning substance use are usually considered confidential, the probability of the outcomes being significant is reduced, despite the anonymity, potentially altering the results.

This study was also carried out among students attending two second-cycle institutions in New Juaben Municipality, an urban community in Ghana. Attempts to generalise the research findings to other students of second-cycle institutions in rural or peri-urban communities may be limited.

The current use of substances was not investigated. Although the prevalence of ever-use is informative, the recent use of substances could be more helpful in determining their menace since many will only use the substance once or a few times.

## Conclusion

To effectively address substance use among high school students, it's essential to incorporate targeted educational initiatives into the school curriculum, particularly for first-year students. Establishing counselling units in secondary institutions can provide crucial support for those struggling with addiction, mitigating potential medical and social consequences. Since stress is a significant factor driving substance use, schools should prioritise recreational activities, support groups and educational sessions focused on stress management. These multifaceted approaches aim to promote healthier choices and support adolescent well-being.

## Supplementary Information

Supplementary Material 1.

## Data Availability

Data and materials are presented in this manuscript and available from the corresponding author upon reasonable request.
