# Supplementary material for: Substance use among Ghanaian adolescents in secondary education: the determinants and medico-social implications
Source: BMC Public Health. 2025 May 2;25:1627. doi: 10.1186/s12889-025-22647-x (PMC12046731; doi:10.1186/s12889-025-22647-x)
Supplement: Supplementary file 1 — Supplementary Material 1. [file 12889_2025_22647_MOESM1_ESM.docx]

**APPENDIX**

**SUBSTANCE USE AMONG GHANAIAN ADOLESCENTS IN SECONDARY EDUCATION: THE DETERMINANTS AND MEDICO-SOCIAL IMPLICATIONS**

This questionnaire is to help us best understand the determinants and medico-social implications of substance use among Ghanaian adolescents in secondary education.
Your participation in the study is voluntary, and we assure you that all information collected will remain confidential.
Please ask when you have a problem understanding a question.

**DEMOGRAPHIC BACKGROUND**

1. What is your age? (In completed years): ……………………………………….
2. Sex/Gender: Male [ ] Female [ ]
3. Religion: Christian [ ] Muslim [ ] Traditional [ ] Others (pls specify)………
4. Form: SHS 1 [ ] SHS 2 [ ] SHS 3 [ ]
5. Program of study: General Science [ ] General Arts [ ] Business [ ] Visual Arts [ ] Technical [ ] Home Economics [ ] Others (pls specify)……
6. Residential status: Boarding [ ] Day [ ]
7. Who do you live with? Both parents [ ] Mother alone [ ] Father alone [ ] Relative [ ] Parents and siblings [ ] Self [ ]
8. Mother’s religion: Christian [ ] Muslim [ ] Traditional [ ] Others (pls specify)…………
9. Father’s religion: Christian [ ] Muslim [ ] Traditional [ ] Others (pls specify)………
10. What is your parent’s/guardian’s occupation? Petty trader [ ] Farmer [ ] Fisherman [ ] Teacher [ ] Doctor [ ] Unemployed [ ] Others (pls specify)…………….
11. Do you work apart from schooling? Yes [ ] No [ ]
12. On an average, how much do you use as pocket money?........................
13. How many siblings do you have? 1-3 [ ] 4-5 [ ] 6-more [ ]
14. What is the occupation of your siblings? Fishing [ ] Farming [ ] Selling/Trading [ ] Others (pls specify)………………
15. What is your source of income? Parents [ ] Guardians [ ] Gifts [ ] Personal work [ ]

**KNOWLEDGE OF SUBSTANCES COMMONLY USED**

1. What substances have you heard of before? Alcohol [ ] Heroin [ ] Tobacco (cigarette) [ ] Marijuana (wee) [ ] Cocaine [ ] Others (pls specify)…………….
2. Have you seen any of these before? Yes [ ] No [ ]
3. If yes, which one? Alcohol [ ] Heroin [ ] Tobacco (cigarette) [ ] Marijuana (wee) [ ] Cocaine [ ] Others (pls specify)……………….
4. Have you used any of them before? Yes [ ] No [ ] (*If no, ignore the next question)*
5. If yes, which one? Alcohol [ ] Heroin [ ] Tobacco (cigarette) [ ] Marijuana (wee) [ ] Cocaine [ ] Others (pls specify)………………….
6. Do you know anyone who has used it before? Yes [ ] No [ ]
7. If yes, who is the person to you? Friend [ ] Schoolmate [ ] Classmate [ ] Parent [ ] Relative [ ] Neighbour [ ] Teacher [ ] Others (pls specify)…………
8. Which of the substances did the person use? Alcohol [ ] Heroin [ ] Tobacco (cigarette) [ ] Marijuana (wee) [ ] Cocaine [ ] Others (pls specify)…………….
9. Does the person still use the drug? Yes [ ] No [ ]
10. If yes, which one? Alcohol [ ] Heroin [ ] Tobacco (cigarette) [ ] Marijuana (wee) [ ] Cocaine [ ] Others (pls specify)…………
11. At what age did you first use the substance? 10-12yrs [ ] 13-15yrs [ ] 16-18yrs [ ] Others (pls specify)…………….
12. Who introduced you to the substance? Friend(s) [ ] Family member [ ] Myself [ ] Drug pushers [ ] Social Media [ ] Others (please specify)…………
13. Do you take more at home or school? Home [ ] School [ ] Both [ ]
14. In the past, have you caused harm to anyone under the influence of some drugs? Yes [ ] No [ ]

**RISK AND PROTECTIVE FACTORS FOR SUBSTANCE USE**

1. Do you have any reasons for using the substances? Yes [ ] No [ ]
2. If yes, what is the main reason? Just enjoy the feeling [ ] To cope with stress [ ] To be like my friends [ ] To improve learning [ ] Others (pls specify)……………….
3. Through what means do you take it? Inject [ ] Sniff [ ] Add to food [ ] Smoke [ ] Others (pls specify)………………….
4. Why did you take your first substance? Curiosity [ ] Parents or relatives offer [ ] Friends encouraged me [ ] To get away from my problems [ ]
5. How can substance use be reduced in schools? Carrying out schools-and communities-based educational campaigns [ ] Enhancing social workers’ ability to raise awareness and detect early signs of addiction [ ] Implementing CCTV systems in schools [ ] Involving religious bodies [ ] Others (pls specify)…………………………. (*tick as many as known)*

**IMPLICATIONS OF THE SUBSTANCE USE**

1. Unable to do homework or study for a test: Yes [ ] No [ ]
2. Got into a fight with other people (friends, relatives, strangers): Yes [ ] No [ ]
3. Caused shame and embarrassment to someone: Yes [ ] No [ ]
4. Neglected duties and responsibilities: Yes [ ] No [ ]
5. Shun by friends and relatives: Yes [ ] No [ ]
6. Missed a day at school: Yes [ ] No [ ]
7. What are some medical consequences of substance use? Psychosis [ ] Stroke [ ] Lung disease [ ] Heart disease [ ] Cancer [ ] Mental illness [ ] HIV/AIDS [ ] Hepatitis [ ] Premature death [ ] Others (pls specify)……….
8. Generally, what effects do substances have on the person? Tiredness [ ] Sickness [ ] Bad temper [ ] Fear [ ] Disturbed sleep [ ] Failure to do well at school/work [ ]
9. What effects do you think substances have on the family? Breakdown in family relationships [ ] Sickness [ ] Disgrace [ ] Loss of trust in child [ ] Worry/fear [ ]
10. What effects do you think substances have on the community? Crime/Violence [ ] Road accidents [ ] Breakdown in work relationship [ ] Reduce productivity [ ]
11. Are you aware that possession and use of substances, specifically narcotic drugs/illicit drugs, in Ghana is an offence? Yes [ ] No [ ]
12. If you have not used any narcotic substances before, do you feel the urge to use them? Yes [ ] No [ ]
13. Do you feel addicted to any of the substances? Yes [ ] No [ ]
14. Will you be willing to be helped if you are addicted to any substance? Yes [ ] No [ ]

***THANK YOU FOR YOUR TIME…***
